# Synthesis and Optimization of Superhydrophilic-Superoleophobic Chitosan–Silica/HNT Nanocomposite Coating for Oil–Water Separation Using Response Surface Methodology

**DOI:** 10.3390/nano12203673

**Published:** 2022-10-19

**Authors:** Syarifah Nazirah Wan Ikhsan, Norhaniza Yusof, Farhana Aziz, Ahmad Fauzi Ismail, Norazanita Shamsuddin, Juhana Jaafar, Wan Norharyati Wan Salleh, Pei Sean Goh, Woei Jye Lau, Nurasyikin Misdan

**Affiliations:** 1 Advanced Membrane Technology Research Centre (AMTEC), Block N29a, Universiti Teknologi Malaysia, Skudai 81310, Johor, Malaysia; 2 School of Chemical and Energy Engineering, Faculty of Engineering, Universiti Teknologi Malaysia, Johor Bahru 81310, Johor, Malaysia; 3 Faculty of Integrated Technologies, Universiti Brunei Darussalam, Bandar Seri Begawan BE1410, Brunei; 4 Faculty of Engineering Technology, Universiti Tun Hussein Onn Malaysia (UTHM), Parit Raja, Batu Pahat 86400, Johor, Malaysia

**Keywords:** sol–gel, chitosan–silica/HNT. hybrid, hybrid nanocomposite, nanoparticle coatings

## Abstract

In this current study, facile, one-pot synthesis of functionalised nanocomposite coating with simultaneous hydrophilic and oleophobic properties was successfully achieved via the sol–gel technique. The synthesis of this nanocomposite coating aims to develop a highly efficient, simultaneously oleophobic-hydrophilic coating intended for polymer membranes to spontaneously separate oil-in-water emulsions, therefore, mitigating the fouling issue posed by an unmodified polymer membrane. The simultaneous hydrophilicity-oleophobicity of the nanocoating can be applied onto an existing membrane to improve their capability to spontaneously separate oil-in-water substances in the treatment of oily wastewater using little to no energy and being environmentally friendly. The synthesis of hybrid chitosan–silica (CTS-Si)/halloysite nanotube (HNT) nanocomposite coating using the sol–gel method was presented, and the resultant coating was characterised using FTIR, XPS, XRD, NMR, BET, Zeta Potential, and TGA. The wettability of the nanocomposite coating was evaluated in terms of water and oil contact angle, in which it was coated onto a polymer substrate. The coating was optimised in terms of oil and water contact angle using Response Surface Modification (RSM) with Central Composite Design (CCD) theory. The XPS results revealed the successful grafting of organosilanes groups of HNT onto the CTS-Si denoted by a wide band between 102.6–103.7 eV at Si^2p^. FTIR spectrum presented significant peaks at 3621 cm^−1^; 1013 cm^−1^ was attributed to chitosan, and 787 cm^−1^ signified the stretching of Si-O-Si on HNT. ^29^Si, ^27^Al, and ^13^H NMR spectroscopy confirmed the extensive modification of the particle’s shells with chitosan–silica hybrid covalently linked to the halloysite nanotube domains. The morphological analysis via FESEM resulted in the surface morphology that indicates improved wettability of the nanocomposite. The resultant colloids have a high colloid stability of 19.3 mV and electrophoretic mobility of 0.1904 µmcm/Vs. The coating recorded high hydrophilicity with amplified oleophobic properties depicted by a low water contact angle (WCA) of 11° and high oil contact angle (OCA) of 171.3°. The optimisation results via RSM suggested that the optimised sol pH and nanoparticle loadings were pH 7.0 and 1.05 wt%, respectively, yielding 95% desirability for high oil contact angle and low water contact angle.

## 1. Introduction

Many recent studies have focused on developing innovative materials with filtering capabilities by instilling them with unique wettability to solve the difficulties of treating wastewater with a high amount of oil and grease [1]. Molecular alteration or material coating has been extensively applied to inorganic substrates, while surface chemistry alteration is typically performed in polymeric materials [2]. Therefore, the concept of a novel matrix with simple functioning and excellent tensile strength is of significant importance [3].

Specifically, the materials with high water affinity and excellent oil repellence have vast potential to be applied in the selective oil-in-water separation process. Therefore, numerous studies have emerged focusing on altering the surface of materials with these properties denoted as simultaneously hydrophilic-oleophobic [4,5,6,7]. One of the most crucial challenges in the treatment of oil-produced wastewater is fouling due to oil adhesion occurrence. Most commercially available filtration materials in the treatment of oil-produced wastewater are commonly made up of only polymer membranes. In terms of its capability, a polymer membrane is still constrained by several drawbacks such as Robeson upper boundary and low fouling resistance [8]. Oil adhesion occurrence is susceptible in polymer membranes due to its wettability. The accumulation of the cake layer due to oil adhesion in the membrane surface has been one of the main factors contributing to the membrane’s fouling.

In mitigating the fouling due to oil adhesion, the membrane should let water through while preventing oil permeation. Thus, the membrane surface needs to be highly hydrophilic and fully oleophobic at the same time. However, the current application of polymer membranes in treating oily wastewater is restricted to one type of wettability that is either hydrophilic or hydrophobic, which disregards the fouling mitigation due to oil adhesion. Attributable to the “uncommon” wettability of water affinity and oil repellence, the superoleophobic/superhydrophilic surface can prevent oil fouling and attain oil in water separation by “water removal” without wetting with water before the application [9]. Chemical alterations of material to achieve oleophobicity are usually related to the modification of surface energy, surface topography, and type of liquid used to influence the wetting.

Therefore, to address the issue brought by polymer membranes, a more flexible and effective modification in ways that can eliminate the occurrence of oil adhesion is required. The membrane material should be simultaneously oleophobic and hydrophilic to ensure spontaneous water separation in an oil emulsion to avoid oil adhesion on the surface. In recent years, mixed matrix membranes have been the common options that were mostly adapted in the industry; however, they require frequent replacement due to oil fouling, making them more costly and inflexible. Moreover, regular filtration shutdowns—to clean the membrane and recover the permeability—increase the costs and complexity of the system. The chemicals used for cleaning the membrane surface also increase the costs and reduce membrane performance and lifespan. As such, the combination of nanomaterials and coating technology can address the issues brought by the current polymer membrane. The developed coating has the desired properties and can also be employed on the existing system that the industry has previously owned.

The application of nanomaterials for wastewater treatment, especially oily wastewater, has been spreading rapidly, and more vigorous research has been conducted concerning the use of nanocomposites. However, finding a suitable hybrid of materials to craft a perfect superwetting and super repellence is very challenging [10]. One approach to accomplishing an industrial-scale treatment of oily wastewater is the proper selection of materials. Efficient superoleophobic adsorbents have previously been developed to be employed as membranes to separate oil and water, enabling one to flow through while repelling the other. This form of the superoleophobic surface has attracted enormous attention with its promising applications in numerous fields, such as the production of non-fogging films, coating with self-cleaning and anti-bioadhesion properties, micro-fluidic systems, and membranes for liquid–liquid separation [11]. The sol–gel coating technique tends to be naturally ubiquitous and provides a tremendous capacity to design products with the ideal morphological characteristics [12].

The use of chitosan (CTS) as a green and inexpensive polymer base in the sol–gel synthesis has been reported in a study by Wang et al. [13]. Chitosan is a type of organic polymer with a positive charge and is, therefore, suitable to be implemented as hydrogel coatings [14] since it contains abundant -NH_2_ [15]. However, due to its high permeability to water, chitosan silica as a coating for water filtration purposes is inefficient due to the increased probability of leaching occurrence. Thus, a more rigid and mechanically stable host is needed for the nanocoating to adhere for water filtration purposes. Halloysite nanotube (HNT), a naturally occurring aluminosilicate nanotube, has been undeservedly forgotten. Halloysite (Al_2_Si_2_O_5_(OH)_4_·2H_2_O) is a two-layered aluminosilicate, with a predominantly hollow tubular structure in the submicron range and chemically similar to kaolin [16]. HNT was found to provide not only mechanical stability and decreasing leaching possibility attributed to its tubular shape, but also improved hydrophilicity of the coating due to its abundant hydroxyl group. Besides that, the arrangement of organosiloxane groups on their inner tube has led to a decrease in the surface free energy, therefore, increasing the oleophobicity of the coating.

Herein, we report on the synthesis of porous, oleophobic, and highly hydrophilic hybrid nanocoating for the oil–water separation membrane by embedding chitosan–silica (CTS-Si) hybrid onto halloysite nanotube (HNT) mixed through a sol–gel process. This oleophobic coating was characterised in terms of morphological and physicochemical properties. The nanocomposite was coated on a polymer substrate to examine its wettability capabilities, and its water contact angle and oil contact angle were calculated by comparing it to the neat substrate and the coating’s derivatives. The resultant nanocoating was then optimised using Response Surface Methodology through the Central Composite Design (CCD) method. The use of a hybrid nanocomposite aiming towards becoming a coating for polymer membranes presents a vast opportunity to alter the existing membrane in the present market to improve their separation capabilities without having to replace the whole system which would contribute towards minimising the development cost.

## 2. Materials and Methods

### 2.1. Chemicals

In this study, the nanocomposite was prepared using a chitosan, silica, and halloysite nanotube. For sol–gel preparation, hydrochloric acid (32% in H_2_O, Sigma Aldrich, St. Louis, MO, USA), ethanol (~0.8g/L, 95%, Fischer Scientific, Waltham, MA, USA), and KH554 (H_4_Si) (99%, Sigma Aldrich) were used as reagents and rinsing agents, while Tetraethyl orthosilicate (TEOS) (98%, Sigma Aldrich) was used as a binder and source of silica.

### 2.2. Synthesis of CTS-Si/HNT Hybrid Sol–Gel Coating

An initial solution of chitosan in 20 mL of water was formed by adding 5.0 g of solid chitosan, 1 mL of concentrated HCl, and 5 mL of ethanol. The mixture was then vigorously mixed until a pale-yellow viscous solution was formed and 5 mL of tetraethoxysilane (TEOS) was then added. Then, 5.0 g of CTS-silica (CTS-Si) gels were dispersed in absolute ethanol, and then 1.0 g of KH554 (H_4_Si, silane coupling agent) was added into the suspension. Modified CTS-Si was obtained after continuous stirring for 24 h at 60 °C, followed by drying in a vacuum oven. The modified CTS-Si was then suspended in water and functionalised onto HNTs by steady dripping. The blends were then sonicated for 30 min to evenly disperse the nanocomposite.

### 2.3. Materials Characterisation

#### 2.3.1. Morphological Characterisation

The morphology of the nanocoating was analysed via Field Emission Scanning Electron Microscopy (FESEM) images on a Hitachi S-4100 (Hitachi, Japan) in the magnification of 2000×. In this analysis, the CTS-Si/HNT hybrid was sputter-coated with platinum to prevent charging, reduce thermal damages, and improve the electron signal needed for the FESEM analysis. The specific surface area of the hybrid is calculated through Brunauer–Emmett–Teller (BET) Surface Area Analysis. Brunauer–Emmett–Teller (BET) analysis was conducted with a specific area and pore analyser (NOVA 2200e, Quantachrome, Boynton Beach, FL, USA). The analysis was conducted using a nitrogen adsorption technique to calculate the total pore volume and surface area of the samples at 77 K using surface area and pore analyser (Anton Paar NovaTouch, Austria). The adsorption data in the relative pressure (P/P0) were used in the range of 0.1 to 0.35 and 0.05 to 0.20, respectively. The total pore volume and the pore-size distributions were computed by applying the Barrett–Joyner–Halenda (BJH) method in the relative pressure (P/P0) range of 0.01 to 0.95.

#### 2.3.2. Cross-Linking and Covalent Union Network of CTS-Si/HNT Hybrid

Nanocomposite and coating patterns and phases were characterised by using X-ray diffraction (XRD: X’pert Pro α1, Philips, Amsterdam, The Netherlands) with Cukα radiation (λ = 1.5406 Å). The diffraction patterns of samples were reported from 10 to 80° 2θ with a phase size of 0.026 and step time of 50 s, worked with a set 1/4° anti-scatter slit at 40 kV and 30 mA. The crystallite size was calculated using Scherrer’s Formula as follows:

$$\tau =\frac{K\lambda }{\beta cos\theta }$$

where *τ* is the mean size of the crystalline, *K* is the dimensionless shape factor of 0.9, *λ* is the X-ray wavelength, *β* is the FWHM, and *θ* is the Bragg angle.

Attenuated Total Reflectance Fourier-Transform Infrared (ATR-FTIR) spectroscopy was used to evaluate the functional group. The specimens used for FTIR measurement were prepared by mixing 0.9 mg of sample powder with 80 mg of KBr and pressing the mixture into a pellet. All samples were studied in the 400–4000 cm^−1^ range, with a resolution of 2 cm^−1^. The experimental setup is similar in the study performed by Massaro et al. (2021) [16]. In situ cross-linking of chitosan and simultaneous formation of CTS-Si/HNT hybrid was revealed by XPS analysis. XPS analysis and high-resolution spectra were documented using Axis Ultra XPS spectrometer (Kratos Analytical, Manchester, UK) at 160 and 20 eV, respectively.

Further cross-linking networking relationship of the nanocomposite was evaluated at the micro-structure level using Solid State NMR. Solid-state 29Si, 27Al, 27Al NMR were analysed on a Bruker Avance III 400 MHz (9.4 T) spectrometer at 79.49 and 100.61 MHz, respectively. ^29^Si MAS NMR spectra were recorded with 4.5 μs 1H 90° pulses, a recycling delay of 60 s and a spinning rate of 5 kHz. ^27^Al CP/MAS NMR spectra were recorded with 3.65 μs 1H 90° pulses, 1.5 ms contact time, a recycling delay of 5 s, and a spinning rate of 9 kHz. Chemical shifts were quoted in ppm relative to tetramethylsilane (TMS). 1H NMR spectra were recorded on Bruker Fourier 300 spectrometer at 300.13 MHz. Deuterated chloroform (CDCl_3_) was used as a solvent, and the chemical shifts are expressed in δ (ppm). Thermogravimetric Analysis (TGA) was performed using modular TGA Q500 to measure the amount and rate of weight change in the hybrid as a function of increasing temperature.

#### 2.3.3. Wettability of CTS-Si/HNT

The wettability of the hybrid was analysed using contact angle through the static sessile drop method, and the free surface energy was assessed using a drop shape analyser DSA100 using paraffin oil for OCA, and distilled water for WCA. CTS-Si/HNT was coated on top of a polysulfone (PSF) polymer membrane as substrate. The contact angle was compared with pristine PSF membrane, CTS-Si-coated PSF, and HNT-incorporated PSF. The membranes were coded following their composition for easier comparison and discussion, as tabulated in Table 1.

#### 2.3.4. Contact Angle Optimisation of CTS-Si/HNT Using Response Surface Methodology

The utilisation of the RSM technique in determining the optimum conditions, namely nanoparticle loading and sol pH towards the coating contact angle, was highly devoted in this study. This is because, apart from the physical properties of the coating, there were external factors that profoundly affect the contact angle of the coating. In this study, it is worth mentioning that the nanoparticle loading and sol pH have imposed a significant impact on the contact angle of the coating. Therefore, the optimisation of the surface contact angle conditions is highly important in this study as a preliminary analysis of the best composition of the nanocomposite and the optimum pH in which the sol will yield a desirable water contact angle and oil contact angle for the application of oil-in-water separation.

The response parameters and the design matrix obtained experimentally with the CTS-Si/HNT nanocoating were tabulated in Table 2. The CCD was performed using two factors full factorial face centred quadratic design. Design-Expert Version 7.0.0 (Stat-Ease, Inc. Minneapolis, MN, USA) software was used for the central composite design (CCD) to understand the factors affecting the water contact angle and oil contact angle of the coating, and to generate the optimised factors that will give the best response. A full factorial design (22) of CCD (face centred) was applied to the two factors with 6 cube points, 4 centre points in a cube, 4 axial points, and a total of 13 runs. Two factors were selected with a low, a central, and a high value, namely loading (mg/mL) and solution pH as they were significant factors that will be affecting the contact angle of the coating. This method was utilised to optimise the contact angle consisting of thirteen runs of the experiment. The quadratic polynomial regression modelling was executed between the two selected independent process variables (nanocomposite loading and sol pH) and the responses (water contact angle and oil contact angle) to attain the best fitted empirical model equation variables and the coded variables. In the confirmation test, the percentage error between the actual to that of predicted data was calculated using the following equation:

Error (%) = (Actual value-Predicted Value)/(Actual value) × 100

## 3. Results and Discussion

### 3.1. Surface Morphology of CTS-Si/HNT Hybrid

#### 3.1.1. FESEM Analysis

**Figure 1:**
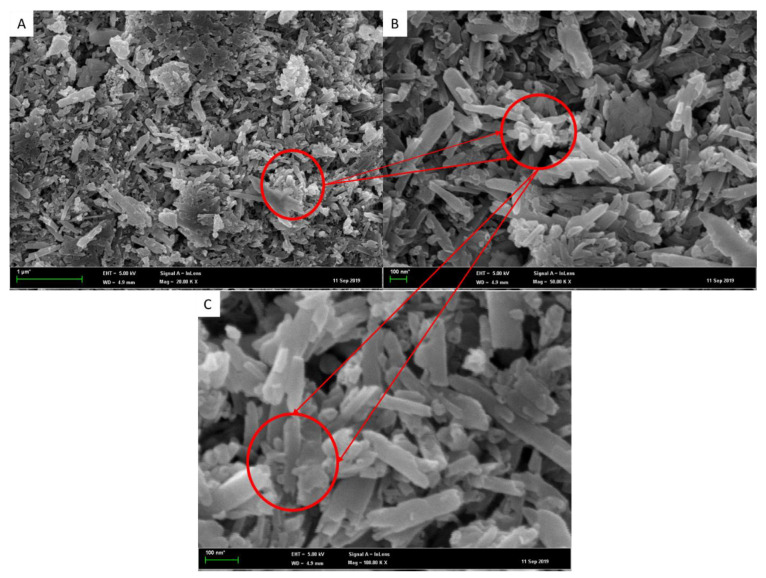
FESEM images of CTS-Si/HNT nanocomposite in (**A**) 20,000×, (**B**) 50,000×, and (**C**) 10,000× magnification.

Figure 1 shows the FESEM image of the CTS-Silica/HNT. nanocomposite in three different magnifications of 20,000, 50,000, and 100,000 times. The 20,000× magnifications in Figure 1A revealed the overall distribution of nanoparticles in the sol–gel. No single-forming chitosan and silica can be found throughout, indicating that all chitosan–silica particles have adhered onto HNT. Upon further magnification of 50,000× in Figure 1B, it can be observed that nanosized spaces and nonuniformly distributed micron-sized hills were overlaid with small HNT tubular bumps. A similar protruding trend can be observed in a study by Li et al. (2020), in which their nanocomposite showed pulling out phenomena due to the interfacial relation between CTS and HNT [17]. The tubular shape of HNT was also found to be thicker and non-symmetric, attributed to the adherence of chitosan–silica, which was further proven through the particle size in XRD and BET analysis that will be discussed in a later section. These discrete organised surfaces have the ability to capture water more efficiently, which helps to create lower contact angles. The silica particles contained in the sol–gel matrix were found to be protruding out of the outer layer as indicated by the red circle in Figure 1C.

#### 3.1.2. BET Analysis

**Figure 2:**
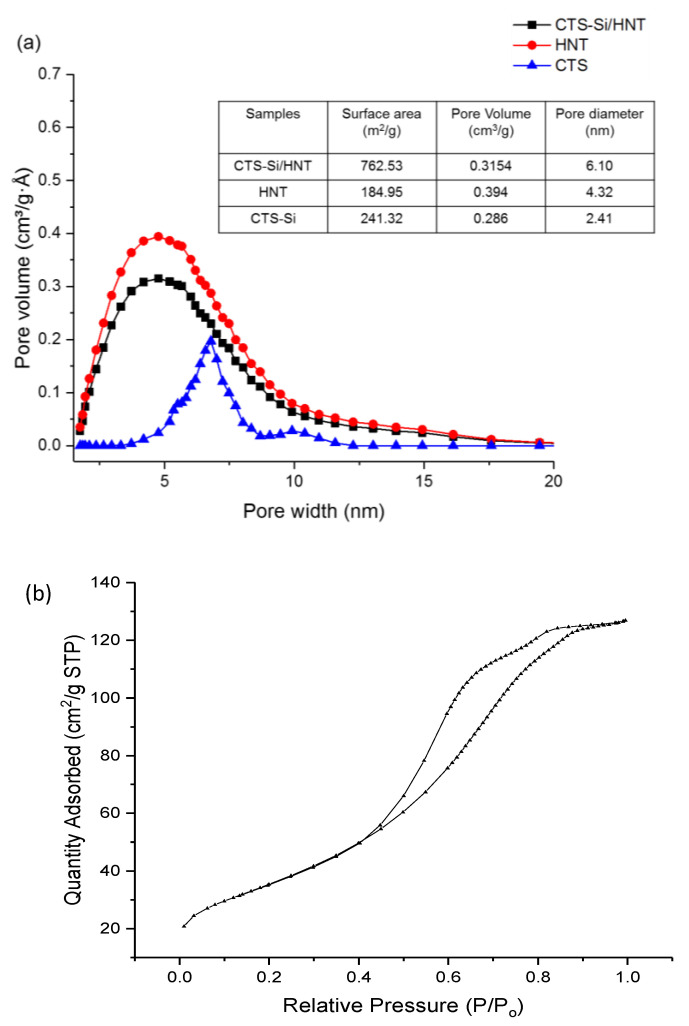
(**a**) Pore distribution of CTS-Si/HNT in comparison to CTS and HNT and (**b**) N_2_ isotherm adsorption of CTS-Si/HNT nanocomposite.

Figure 2a shows the pore width distribution of the nanocomposite. From the analysis, it has been deduced that the calculated surface area, pore-volume, porosity, and pore diameter were 762.53 m^2^/g, 0.3154 cm^3^ g^−1^, 0.1911 nm^3^ g^−1^, and 6.10 nm, respectively. Therefore, the porosity of the resultant nanocomposite was categorised as microporous (pore diameter less than 2 nm), which agrees with the adsorption isotherm type IV that is shown in Figure 2b. This result also confirms the nanocomposite’s high surface-area-to-volume ratio, which contributed to its enhanced oleophobicity [18].

Type IV isotherms describe the adsorption behaviour of special mesoporous materials showing pore condensation together with hysteresis behaviour between the adsorption and the desorption branch. These characteristics are often found in highly hydrophilic materials [19]. Moreover, the N_2_ isotherm shape showed a concave-shaped curve which denotes the existence of micro pores with available surface area found mostly in the inner side of the pores. Adsorption will be stopped once these pores are filled, which creates a plateau region in the BET curve. The plateau region indicates the increased surface area, which correlates directly with the improved water adsorption.

### 3.2. Functional Group and Phase Composition of CTS-Si/HNT Hybrid

#### 3.2.1. FTIR Analysis

**Figure 3:**
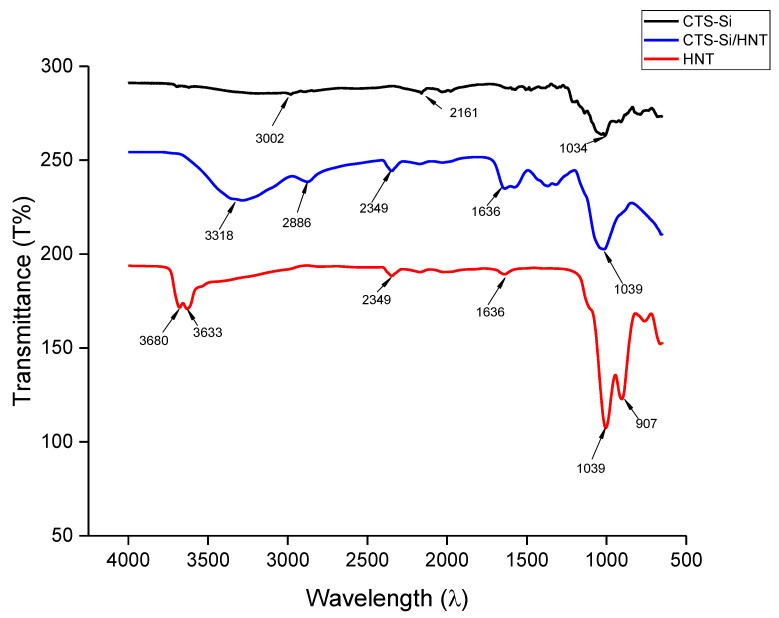
FTIR spectra of CTS-Si/HNT nanocomposite in comparison with CTS-Si and HNT.

The symmetric vibration of free NH_2_ and OH groups is visible in all FTIR spectra indicated by a wide band in the range of 3200 to 3600 cm^−1^ which originate mostly from chitosan and silanol group in silica [20]. Figure 3 showed that multiple significant peaks attributed to different functional groups in the nanocoating. The absorption characteristics of chitosan with OH stretching were found at 3002 cm^−1^, C-H at 2161 cm^−1^, and C=O at 1034 cm^−1^. It can be observed that the O-H stretching at CTS-Si/HNT has become stronger and broader, indicating the increased amount of hydroxyl group in the nanocomposite, which attributes to its increased hydrophilicity. The peak at 907 cm^−1^ observed only in HNT FTIR spectra is attributed to Al-OH bonding which does not appear in CTS (due to absence of Al) and also disappeared in CTS-Si/HNT nanocomposite due to the breaking of Al-OH bonding after when CTS-Si is functionalised onto HNT through H-bonding. Similar result has been showed through study by Zhang and Wu (2018) [21] and Zeng et al., (2017) [22].

Meanwhile, the characteristic of Si-O band specified at 1000 to 1100 cm^−1^ was observed, in line with the result reported by Lakshmi et al. (2014) [23]. The double peaks at 3680 cm^−1^ and 3633 cm^−1^ were observed due to the stretching vibration of the hydroxyl group at the surface of HNTs. The attachment of CTS-Si to HNT can be denoted by the C-H stretching that occurred at 2886 cm^−1^ to 2349 cm^−1^ region and overlaps the vibration from the carbohydrate ring. The symmetric stretching of Si-O-Si at 1039cm^−1^ has further confirmed the incorporation of CTS-Silica on HNT. The widened peaks from 1100 cm^−1^ to 1200 cm^−1^ belonged to Si-O-Al and Si-O-Si groups, and in comparison with HNT, the peak was divided into two, but in CTS-Si/HNT the peak is combined as one and is wider, which denoted to the apparent bonding of KH550 on the inner surface of Al-OH groups which is in agreement with the finding reported by Doermbach et al. (2013) [24].

#### 3.2.2. XRD Analysis

**Figure 4:**
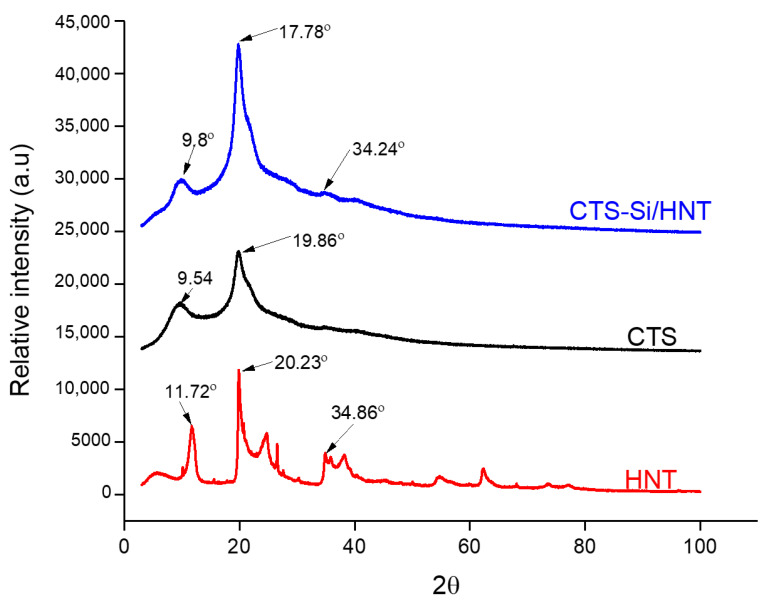
XRD diffraction of CTS-Si/HNT Nanocomposite, CTS-Si, and HNT.

The analysis of XRD diffraction of CTS-Si/HNT nanocomposite, CTS-Si, and HNT are depicted in Figure 4. In the CTS-Si/HNT spectra, only three apparent peaks can be observed, which match the three prominent peaks of HNT at 2θ = 9.8, 17.78, and 34.24. Meanwhile, the two characteristic peaks can be observed in CTS-Si spectra, at 2θ = 9.54 and 19.86, which is the characteristic diffraction peak of chitosan. Both CTS-Si and CTS-Si/HNT have a similar angle of the spectrum from 2θ = 40 onwards, indicating that the crystalline form was disrupted due to the interaction of chitosan and silica, which caused the region to become broad. The basal reflection at around 2θ = 11.72° assigned to a (1 0 0) plane with d-spacing of 0.877 nm indicated that the HNT multiwall tubular structure is merged into one peak at 2θ = 9.8° on CTS-Si/HNT. The basal reflection at around 2θ = 9.8 was assigned to a (1 0 0) plane with d-spacing of 0.877 nm, indicating that the HNT multiwall tubular structure was present at both CTS-Si/HNT and HNT spectra, but not on CTS-Si.

The slight peak at 2θ = 34.24 was assigned to (4 1 0) plane with d-spacing of 0.454 nm, indicating the presence of the Al-Si group, which was apparent in HNT spectra at 2θ = 34.86 but absent in CTS-Si. Peak shift for CTS-Si/HNT is at 2θ = 17.78 at the plane (2 1 0) with d-spacing = 0.438 nm, in comparison to those of the HNT spectra, in which the sharp peak is located at 2θ = 20.23 with d-spacing = 0.33 nm. This peak shift indicates the intercalation of CTS-Si onto the hydroxyl surface of HNT, which correlates with the result from FTIR spectra.

#### 3.2.3. XPS Analysis

The key elements in HNTs were recorded as oxygen, aluminium, and silicon with an Si:Al ratio of 1 to 1, which is compatible with the usual two-layer (1:1) aluminosilicate structure, as shown in Table 3. As the HNT functionalised with the chitosan–silica hybrid, the Si/Al ratio increased to 1:6 as the major components (oxygen, aluminium, and silicon) with Si/Al ratio (from 1.0 to 1.6). This successful functionalisation is further evidenced by the increasing amount of the surface carbon percentage and additional target components, for example, nitrogen. The change in the Si/Al ratio and the rise in the C surface composition, and the emergence of additional elements attributed to organosilanes indicated the success of the organosilane attachment on the anterior and posterior of HNT surfaces [25].

**Figure 5:**
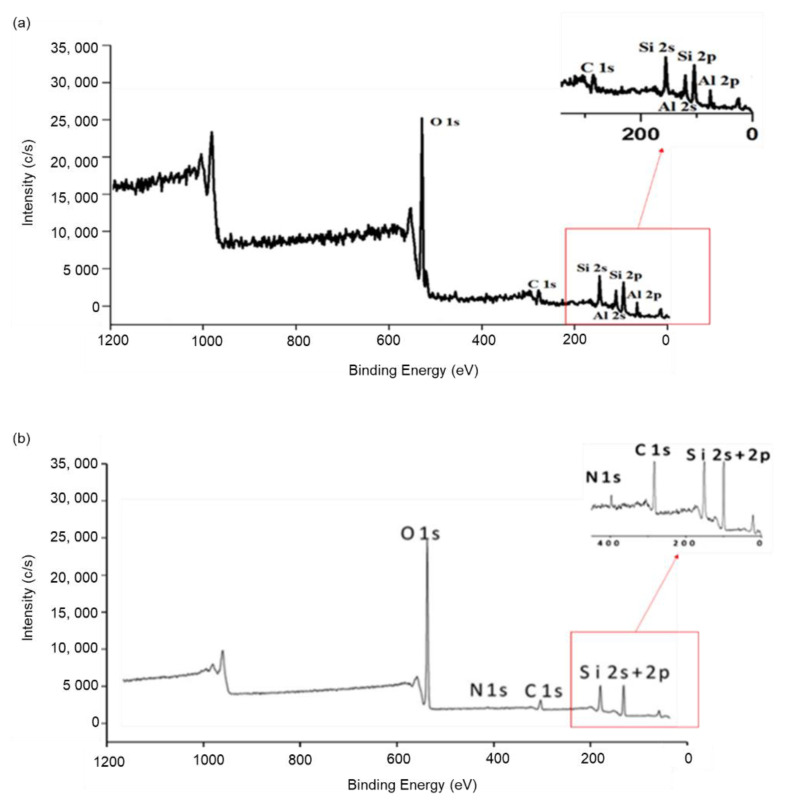

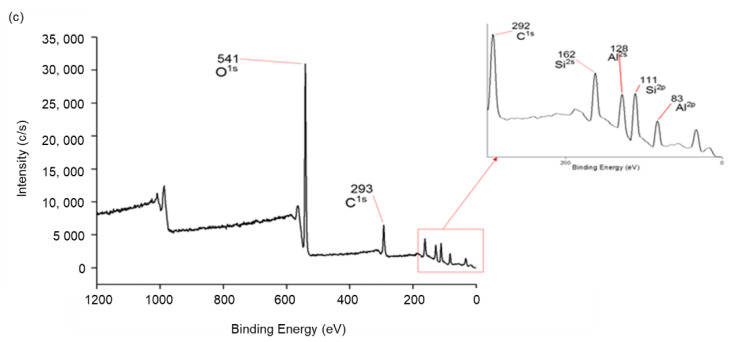
XPS scan of (**a**) HNT., (**b**) CTS-Si, and (**c**) CTS-Si/HNT.

As depicted in Figure 5, in the C1s region, the CTS-Si/HNT spectrum has a peak at 293 eV, denoting N-H bonds of free amine groups [26]. These peaks are more intense (5014 c/s) in comparison to HNT (4589 c/s) and CTS-Si (4887 c/s). The peak for silicon at 111 eV is due to the cyanite ester group of -OCN on the HNT side of the nanocomposite [27]. This peak also corresponds to the Si-O bond, similar to findings by Fu et al. (2020) [28]. The spike for silicon at 108.2 eV in HNT from Figure 5a is suggested for silicon in silanols or Si-O-Si. This peak was found to split into a new peak at 111.12 eV for CTS-Si/HNT from Figure 5c, which is due to the association of silicon in the CTS-Si hybrid amino carbonate. In addition, Al2p position in CTS-Si/HNT shifted to a lower region (83 eV) in comparison to HNT (94.5 eV) due to the reaction of silane coupling agent between CTS-Si and the outer surface of HNT, which is in accordance with the study conducted by Paran et al. (2016) [29].

Interfacial reactions which resulted in inorganic tubes that are linked covalently with the matrix are what is responsible for the decreased C1s composition, increased modulus, and altered morphology of the hybrids [30]. Furthermore, the Si 2p area reveals a wide band in the range of 102.6–103.7 eV in both HNT and CTS-Si/HNT as seen in Figure 5, indicating the presence of Si-O bonds not only due to the inherent nature of HNTs but also because of functionalised organosilanes in the case of CTS-Si/HNTs [31,32]. These results were in agreement with the ^29^Si NMR analysis, which further investigates the cross-linking of chitosan–silica on the surface of HNT that will be explained in a subsequent section.

#### 3.2.4. NMR Analysis

**Figure 6:**
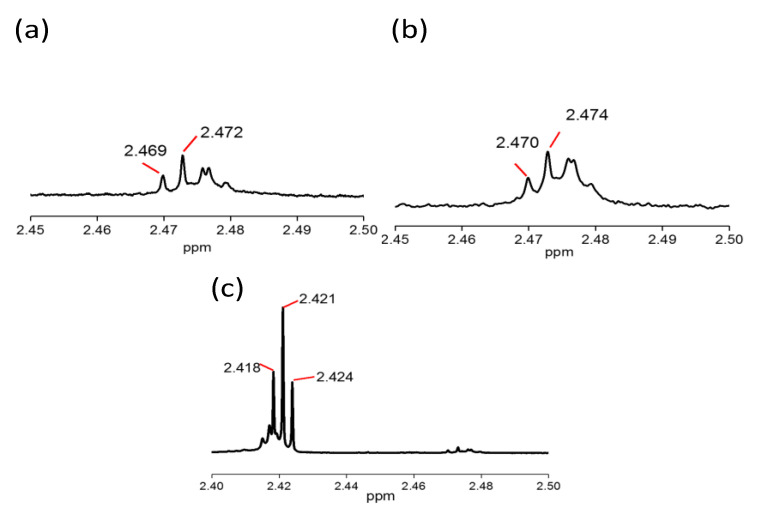
1H NMR of (**a**) HNT, (**b**) CTS, and (**c**) CTS-Si/HNT.

The reaction on the cross-linking was evidenced with the peaks in 1H NMR for CTS-Si/HNT and is shown in Figure 6 where it can be seen that 1H NMR of HNT in Figure 6a and CTS in Figure 6b have double peaks at around 2 to 2.5 ppm, attributed to the unsaturated HCN and C=C groups. However, 1H NMR of CTS-Si/HNT in Figure 6c shows a down field chemical shift to the right which shows the reaction on the crosslinking of HCN from Chitosan and C=C from HNT. In addition to that, the peak was split into triplet, detected at 2.418, 2.421, and 2.424 ppm, attributed to the reaction of the acetyl group in Chitosan-Si onto HNT due to the hydrolyzation and condensation of all three ethoxyl groups. In addition, the pattern in which the triplet split shows a first order split, indicating that there was an increase in double bond formation through geminal proton-proton coupling of HCN and C=C between CTS-Si and HNT.

**Figure 7:**
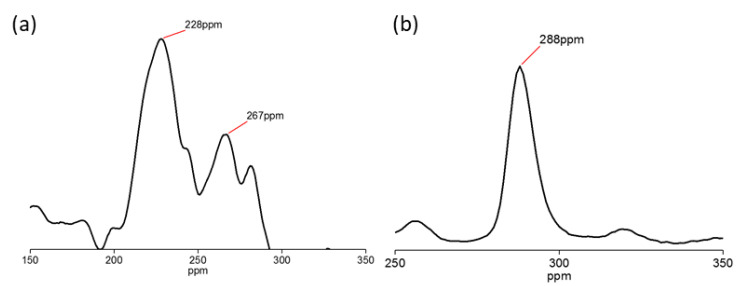
27Al NMR of (**a**) HNT and (**b**) CTS-Si/HNT.

This is supported by the result recorded in ^27^Al NMR shown in Figure 7, where the peak for Al in pristine HNT was detected at 228 ppm (Figure 7a), and the peak shifted to 288 ppm in CTS-Si/HNT, attributed to the shifting of Al-Si bonds as shown in Figure 7b. Similar results were also reported by previous research conducted on HNT and chitosan–silica hybrids, respectively [33,34]. This indicates that the shift in the Al-Si group on the surface of HNT was in accordance with a previous study [35] which was due to the crosslinking with chitosan–silica hybrid on the external surface of HNT, which was similar to other findings [36].

**Figure 8:**
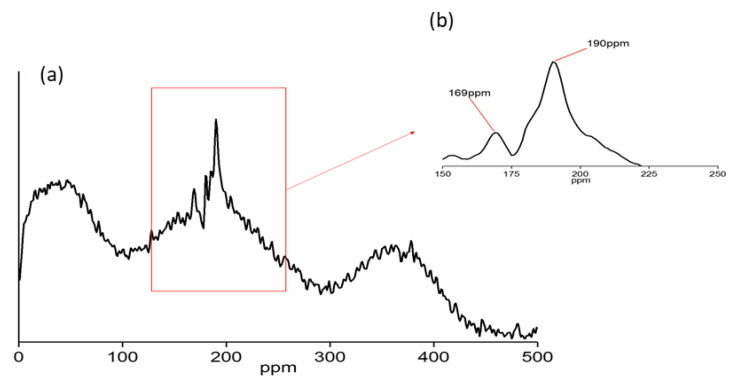
29Si NMR of CTS-Si/HNT where (**a**) peak at 500 ppm magnification and (**b**) peak at 200 ppm magnification.

On the other hand, the ^29^Si NMR showed that the hydrolysis of two Si–O–Al and two Al–OH–Al bonds occurred during the release of single Al^3+^ ions due to the crosslinking between CTS-Si onto HNT via the coupling agent, as shown in Figure 8a. These were evidenced by two newly formed broad chemical shifts at approximately 169 ppm assigned to the Si-O-Si-Al-H and 190 ppm assigned to Si-O-Si sites in CTS-Si/HNT as shown in Figure 8b, which is similar to those performed by [36]. The combination of ^29^Si and ^27^Al NMR spectra indicates that there were hydrolyzation reactions at the Al–OH groups (inner lumen surface and edges) and Si–OH groups from CTS-Si at the edges or external surface of HNT [37].

### 3.3. Colloidal and Thermal Stability of CTS-Si/HNT Hybrid

**Figure 9:**
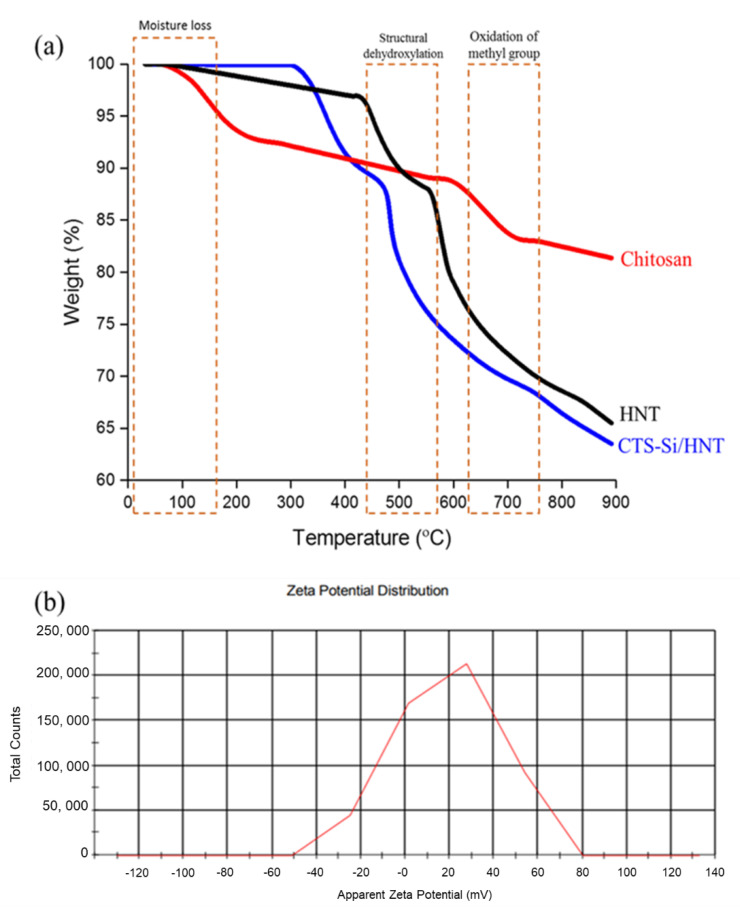
(**a**) TGA analysis of CTS-Si/HNT in comparison with HNT and Chitosan and (**b**) apparent zeta potential of CTS-Si/HNT.

TGA analysis was performed on HNT, CTS-Si, and CTS-Si/HNT to evaluate the effects of HNT hybridisation on CTS-Si towards the nanocomposite’s thermal stability. There were three main decomposition steps for the nanocomposite. As shown in Figure 9a, in the first stage, there was 58.6% weight loss from temperatures of 290 °C to 320 °C, due to carbohydrate-backbone fragmentation [38]. The first stage represented the physical evaporation of water that was adsorbed on the composite surface, while the second stage depicted the carboxyl and hydroxyl condensation [39].

CTS-Si/HNT’s colloidal stability is calculated by zeta potential analysis. Zeta potential is the difference of the electrostatic potential between the substrate and the liquid layer connected to the suspended particles of the substrate, where the zeta potential indicates the degree of electrostatic repulsion between the immobilised nanoparticles [40]. Weak repulsive forces occur amongst the nanoparticles due to low zeta potential leading to aggregation of the particles resulting from the Van der Waals attraction [41]. In addition, colloids lose stability and agglomeration when the pH is close to the isoelectric point. However, at low zeta potential, the ionic strength of nanoparticles increased, and it becomes more compact [42]. From the distribution graph shown in Figure 9b, the general zeta potential of the colloids is in the positive charge range. The specific measured zeta potential for the resultant CTS-Si/HNT is 29.3 mv, reflecting on the stability of the hybrid colloids. This result is in line with the electrophoretic mobility of the dispersed colloids recorded at 0.1904 µmcm/Vs, indicating stability due to the small magnitude of mobility [43].

### 3.4. Wettability of CTS-Si/HNT Hybrid

#### 3.4.1. Water Contact Angle

**Figure 10:**
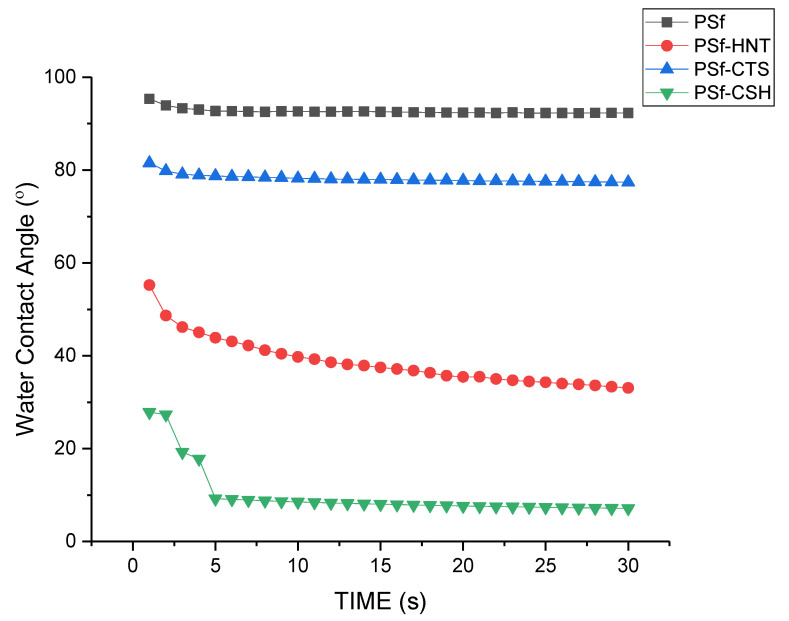
Dynamic contact angle of different types coating onto PSF substrate.

Figure 10 shows the dynamic water contact angle (WCA) of different types of nanocomposite coating onto PSF substrate, in which the contact angle was recorded for 30 s, with a 1 s frame interval. It can be seen that for the neat substrate, the contact angle started quite high at 96.5°, and there was only a slight reduction after 30 s, to 85°, which represents slight hydrophilicity. A remarkable difference can be seen in PSF-HNT, in which the water contact angle started very low, at 54°, and continued to drop rapidly to the final contact angle of 11° at the 30 s mark. This difference shows rapid diffusion of water into the membrane, indicating superhydrophilicity [44]. One apparent cause for this improvement of hydrophilicity is the structure of the nanocoating which is porous due to the network form between halloysite nanotubes as it has a lower percolation threshold [45]. A drop in water contact angle can be observed in PSF-CTS, due to hydrophobicity resulting from the silica group in CTS-Si. However, a significant decrease in WCA can be seen in CTS-Si/HNT-incorporated membrane, which is majorly contributed to by HNT that overrides the slight hydrophobicity imbued by CTS-Si. Therefore, it is evident that the addition of CTS-Si/HNT onto PSF has improved its hydrophilicity by decreasing the dynamic water contact angle significantly through the interaction between HNT and CTS-Si at the surface of the membrane.

#### 3.4.2. Oil Contact Angle

**Figure 11:**
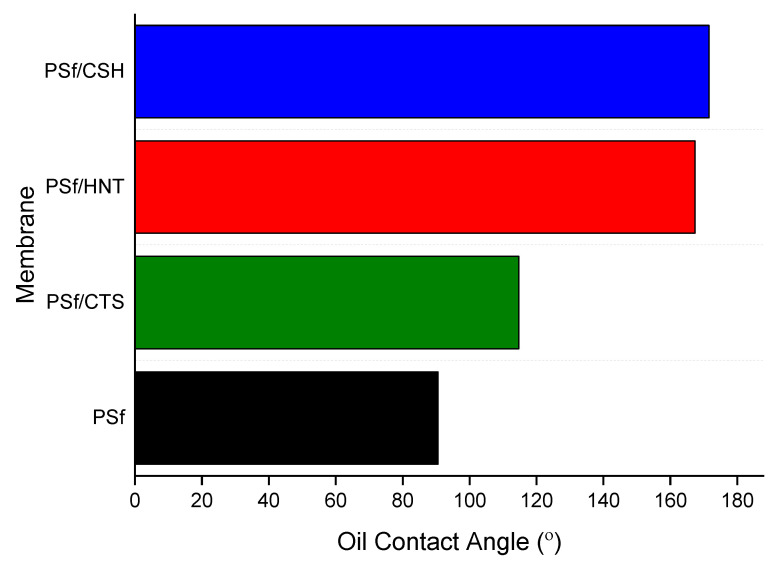
OCA for membranes incorporated with different types of nanocomposites.

Oleophobicity of the nanocomposite was analysed and compared similarly to hydrophilicity analysis by replacing water with oil in the contact angle analysis. The oil contact angle was performed using sessile drop using paraffin oil to replace water drop. The result of the static oil contact angle was tabulated in Figure 11. A significant increase in the contact angle was observed from 43.6° for PSF to 121.5° for PSF-HNT. A further increment of OCA was observed in PSF-CTS (154.2°). The PSF-CTS membrane recorded the highest oil contact angle of 171.3°, attributed to the increased amount of organosilane in the coating as analysed by XPS in the previous section. In addition to that, the calculation of the volume-to-surface-area ratio is also high, as mentioned in the BET analysis, which also contributes to the high oil contact angle, reflecting the higher oleophobicity of PSF-CTS. Subsequently, the nanocoating CTS-Si/HNT has significantly increased the surface oleophobicity of the PSF membrane.

### 3.5. Response Surface Methodology Model Analysis

#### 3.5.1. Oil Contact Angle Responses

The evaluation of the significance of mathematical regression model fitness was confirmed by running the experiments on the model suggested by the CCD. Apart from the fitness, the model coefficients and lack of fitness calculated using variance (ANOVA) analysis using the second-order response model were performed. The data obtained from the ANOVA analysis of the oil contact angle concerning the loading and sol pH is summarised in Table 4. The regression results were associated with some model terms, which explained the model adequacy and significance.

From the results obtained, it showed that the Model F-value of 24.92 implies the model is significant. There is only a 0.03% chance that a “Model F-Value” this large could occur due to noise while the experiments are running. Values of “Prob > F” less than 0.0500 indicate that the model terms are significant. The *p*-value was also used to check the significance of each coefficient, and the smaller the p-value was, the more significant the corresponding coefficient was. It represents a decreasing index of the reliability of a result and the probability of error involved in accepting the observed result as valid. In this case, all factors, namely loading (A) and sol pH (B), and the interaction between them, A^2^, B^2^, and AB are significant model terms.

The “Lack of Fit F-value” of 2.26 implies there is a 22.30% chance that a “Lack of Fit F-value” this large could occur due to noise. From the calculated data, all factors manipulated in this model affect the coating contact angle, with all factor interaction values denoted as significant. The regression model is statistically significant if *p* < 0.0500 at 95% confidence level. The non-significant lack of fit F-value indicates that the model has good experimental data fitting. The lack of fit measures the model’s failure to represent data in the experimental domain at points that are not included in the regression [46].

An adequate fit of the model should be obtained to avoid poor or ambiguous results in optimising a response surface. This is important to ensure the adequacy of the employed model. Table 5 shows the summary of regression parameters of the predicted response surface quadratic model for oil contact angle using all the results of all experiments performed. The ratio of 12.88 indicates an adequate signal for the model. The predicted R-squared of 0.75 is in reasonable agreement with the Adj R-Squared of 0.91. The value of the correlation coefficient (R^2^ = 0.95) indicates that the empirical model could not explain only 5% of the total variation and expresses good enough quadratic fits to navigate the design space.

The value obtained in the present study for these response variables was higher than 0.80, indicating that the regression models explained the reaction well. Hence, the response surface model developed in this study to predict the oil contact angle of the nanocoating was considered satisfactory.

**Figure 12:**
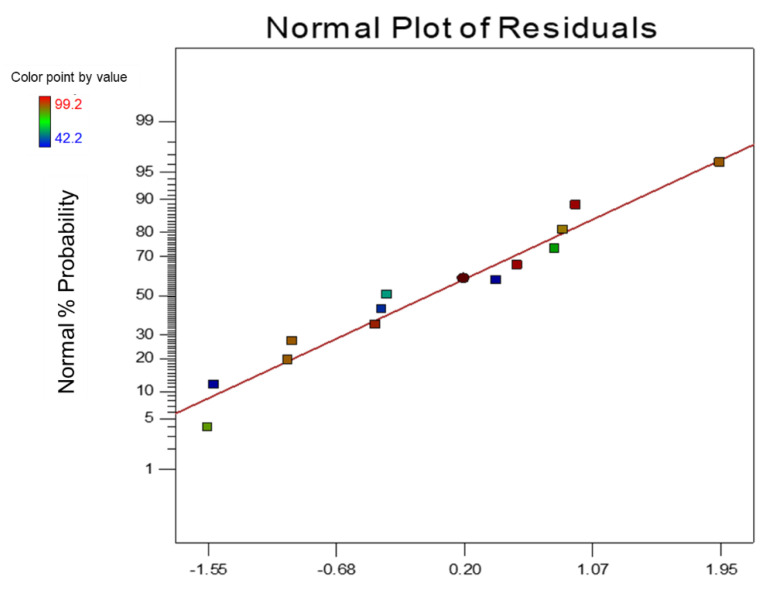
Normal Probability Plot of Oil Contact Angle Optimisation.

The analysis of the normal probability of the residual of the oil contact angle is depicted in Figure 12. From the figure, it can be seen that the normal probability formed a nearly straight line of the residual distribution. The normal probability plot of the residual oil contact angle indicates a significant number of data that formed a straight line, with a small amount of departure from the distribution—the data points clustered around the straight line with normalised skewing on both sides. Denoting errors are evenly distributed, indicating an adequate and reasonable model which is free from variance.

#### 3.5.2. Water Contact Angle Response

In this section, a similar CCD setup was employed to establish the relationship of both loading and sol pH and the water contact angle of the nanocoating. The water contact angle response reflects the contact angle’s hydrophilicity, which can affect the flux during the filtration experiment. It is trivial that to draw the line at least two points are required, and for a quadratic curve, at least three points are required. Hence, the experiment was performed for at least three levels of each factor to fit a quadratic model. This method is suitable for fitting a quadratic surface and helps to optimise the effective parameters with the minimum number of experiments and analyse the interaction between parameters. The data on ANOVA evaluation of water contact angle response is tabulated in Table 6.

From the data, it showed that the Model F-value of 12.11 implies the model is significant. There is only a 0.25% chance that a “Model F-value” this large could occur due to noise while the experiments are running. Values of “Prob > F” less than 0.0500 indicate that model terms are significant. The *p*-value was also used to check the significance of each coefficient, and the smaller the *p*-value was, the more significant the corresponding coefficient was. It represents a decreasing index of the reliability of a result and the probability of error involved in accepting the observed result as valid. In this case, all factors, namely loading (A) and sol pH (B) and the interaction between them, A2, B2, and AB, are significant model terms.

The “Lack of Fit F-value” of 2.58 implies there is a 19.12% chance that a “Lack of Fit F-value” this large could occur due to noise. From the calculated data, all factors manipulated in this model affect the coating contact angle, with all factor interaction values denoted as significant. The regression model is statistically significant if *p* < 0.0500 at 95% confidence level. The non-significant lack of fit F-value indicates the model has good experimental data fitting. The lack of fit measures the model’s failure to represent data in the experimental domain at points that are not included in the regression.

Further evaluation of the optimised response surface is conducted using R-squared, and the values of coefficient of determination (R^2^) and adjusted coefficient of determination are 0.90 and 0.82, respectively, which are close to unity and show high conformity between actual and predicted results. R^2^ values of 0.90 indicate that only 10% of the total variation could not be explained by the empirical model and expresses good enough quadratic fits to navigate the design space. Adequate precision measures the signal to noise ratio with a ratio higher than 4 signify a desirable ratio. The ratio of 8.71 indicates an adequate signal so that this model can describe the response behaviour properly, based on the independent variables.

**Figure 13:**
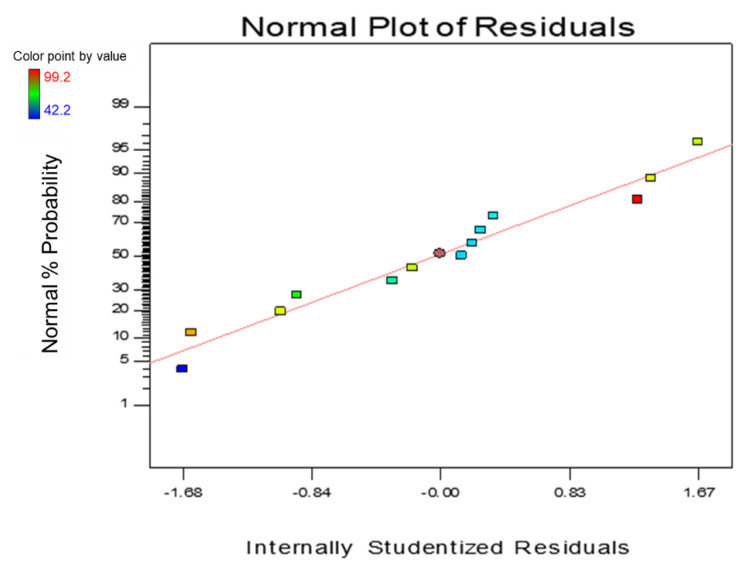
Normal plot of residuals for water contact angle optimisation.

The analysis of the normal probability of the residual of the oil contact angle is depicted in Figure 13. The normal probability plot of the residual water contact angle indicates a significant number of data that formed a straight line, with a small amount departure from the distribution—the data points clustered around the straight line with normalised skewing on both sides. Denoting that errors are evenly distributed, indicating the adequate and reasonable model which are free from variance.

#### 3.5.3. Optimisation of Confirmatory Test

The confirmatory test was performed to verify the adequacy of the developed model. In this study, five confirmation tests were performed to evaluate the model by comparing the response equations and the suggested predicted values to the experiments’ actual values. The comparison data of the actual over the predicted values of both responses (oil contact angle and water contact angle) are tabulated in Table 7. The correlation shows that there was good agreement between the actual and predicted data with 1.61% and 1.91% average errors for oil contact angle and water contact angle, respectively. Moreover, the obtained value of average errors less than 5% indicated that the model adequacy was reasonable at 95% of the prediction interval.

## 4. Conclusions

The efficient and effective functionalisation of CTS-Si on HNT has been demonstrated by substantial physicochemical characterisation, which involves morphological, structural, textural, chemical, and spectroscopic methods. The resultant nanocomposite showed the highest oil contact angle, which corresponds directly to its oleophobicity attributed to the increased organosilane group in the nanocomposite coating. The hydrophilicity of the nano coating has also shown significant improvements attributed to the increased amount of -OH groups and increase in surface area of the nanocoating. Consequently, the surface charge of the coated membrane has also affected its wettability in which the hybrid coating recorded the lowest water contact angle of less than 10° in comparison to its derivative. The oil contact angle was also recorded as high, whereas the difference with HNT was almost the same, suggesting that the oleophobicity was mostly contributed to by the abundance of organosiloxanes in HNT. The excellent simultaneously hydrophilic-oleophobic nanocoating synthesised in this study has tremendous potential to be applied in oily wastewater treatment due to its favourable characteristics of being green, cost-effective, and having a simple operation. Additionally, the optimisation was successfully performed using the design expert software with face centred central composite design assisted by the ANOVA analysis. The finding has suggested that the nanocomposite loadings play a more significant effect to the coating contact angle in contrast to the sol–gel pH. The optimum condition for the coating to have the best water contact angle and oil contact angle was found to be at 1.05 mg/mL nanocomposite loading and 7.5 sol–gel pH. The confirmatory test has revealed that the optimum performance was acceptable with average errors of 1.91% and 1.78% for the water contact angle and oil contact angle, respectively. Furthermore, the optimisation analysis suggested that the studied formulation of the nanocoating hybrid was at its optimum capacity that can result in desirable wettability, which can be highly efficient for the application of oil-in-water separation, especially those that require low energy and a high-performing membrane.

## 5. Future Outlooks and Recommendations

From the results obtained in this study, there are some recommendations and suggestions for the improvement of this study for future applications. First, it is recommended that filtration studies are performed for the removal of other potential contaminants using the synthesised nanocomposite coating. This type of coating might be useful for the treatment of highly toxic contaminants such as the removal of heavy metals or brackish water treatment. In this case, further studies on the different operating conditions for these applications are needed to ensure the versatility of the synthesised nanocomposite coating. In addition to that, industrial scale operation conditions such as oily wastewater from different sources (refinery, palm oil mill, offshore drilling rig), high temperature, and pressure should also be investigated, and a longer filtration period should also be carried out to investigate the applicability of the coating on real-life processes in harsh conditions. Such investigations should be conducted to assess its capability and readiness to be scaled up for industrial-scale processes. Finally, oil reclamation and recovery application development is needed for future research in relation to this study. The nanocomposite coating is not only applicable for the treatment of oily wastewater but has shown potential to be used as a means for improving oil reclamation processes. Therefore, further studies on improving the quality of the retentate instead of only permeation during filtration should also be investigated to ensure the capability of the coating for this process.

## Figures and Tables

**Table 1:** Differently modified PSF substrates for contact angle analysis.

Membrane Code	Substrate	Coating/Additives
PSF	Polysulfone polymer membrane	Neat Substrate
PSF-CTS		CTS-Si/HNT
PSF-HNT		HNT
PSF-CTS		Chitosan

**Table 2:** CCD design matrix of 2^2^ factorial and experimental responses.

Run	Factor 1	Factor 2	Response 1	Response 2
	Loading (mg/mL)	pH	OCA (°)	WCA (°)
1	1.5	7.5	173.21	64.95
2	2	5	135.42	110.29
3	1	5	125.71	94.35
4	0.79	7.5	171.21	72.39
5	1.5	3.9	115.78	100.69
6	2.21	7.5	147.43	92.67
7	2	10	122.22	94.25
8	1.5	7.5	183.83	64.49
9	1.5	7.5	186.55	65.89
10	1	10	164.84	83.121
11	1.5	10	173.39	92.321
12	1.5	7.5	177.25	52.31
13	1.5	7.5	173.42	65.32

**Table 3:** Surface atomic percentages determined by XPS of CTS-Si/HNT., HNT., and CTS-Si.

Material	C1s	O1s	Si 2p	Al 2p	N1s	Si/Al
HNT	5.8	64.0	15.6	14.7	-	1.1
CTS-Si	68.95	23.20	7.00	-	0.85	-
CTS-Si/HNT	29.3	46.2	12.5	8.9	2.6	1.6

**Table 4:** Analysis of variance (ANOVA) for oil contact angle response.

Source	Sum of Squares	df	Mean Square	F Value	*p*-Value	
					Prob > F	
Model	7106.45	5	1421.29	24.92	0.0003	significant
A-Feed pH	553.48	1	553.48	9.7	0.017	
B-Dipping time	492.52	1	492.52	8.63	0.0218	
AB	684.76	1	684.76	12	0.0105	
A2	1059.3	1	1059.3	18.57	0.0035	
B2	3507.65	1	3507.65	61.49	0.0001	
Residual	399.31	7	57.04			
Lack of Fit	251.35	3	83.78	2.26	0.223	not significant
Pure Error	147.96	4	36.99			
Cor Total	7505.76	12				

**Table 5:** Summary of ANOVA and regression analysis of oil contact angle.

Response Surface	Quadratic Model
R-Squared	0.95
Adj R-Squared	0.91
Predicted R-Squared	0.75
Adequate Precision	12.88

**Table 6:** Analysis of variance (ANOVA) for water contact angle response.

Source	Sum of Squares	df	Mean Square	F Value	*p*-Value	
					Prob > F	
Model	3379.26	5	675.85	12.11	0.0025	significant
A-Loading	388.49	1	388.49	6.96	0.0335	
B-pH	22.33	1	22.33	0.40	0.5471	
AB	5.78	1	5.78	0.10	0.7570	
A^2^	709.87	1	709.87	12.72	0.0091	
B^2^	2099.48	1	2099.49	37.62	0.0005	
Residual	390.69	7	55.81			
Lack of Fit	257.52	3	85.84	2.58	0.1912	not significant
Pure Error	133.17	4	33.29			
Cor Total	3769.95	12				

**Table 7:** Confirmatory Test for Optimum Responses of OCA and WCA.

Run	Oil Contact Angle			Water Contact Angle		
	Actual	Predicted	Error (%)	Actual	Predicted	Error
1	179.2	175.52	2.05	56.2	54.33	3.33
2	181.8		3.45	55.21		1.59
3	176.9		0.78	54.95		1.13
4	177.3		1.00	55.21		1.59
5	178.4		1.61	55.39		1.91
Average error			1.78	Average error		1.91
Standard deviation			0.949501	Standard deviation		0.75

## Data Availability

The data presented in this study are available in the article and Supplementary Materials.

## Supplementary Materials

The following supporting information can be downloaded at: https://www.mdpi.com/article/10.3390/nano12203673/s1, Table S1: The crystalline information of each nanoparticle; Table S2: TGA data of CTS-Si/HNT nanocomposite in air.
