# Supplementary material for: Synthesis and Optimization of Superhydrophilic-Superoleophobic Chitosan–Silica/HNT Nanocomposite Coating for Oil–Water Separation Using Response Surface Methodology
Source: Nanomaterials (Basel). 2022 Oct 19;12(20):3673. doi: 10.3390/nano12203673 (PMC9607117; doi:10.3390/nano12203673)
Supplement: Supplementary file 1 [file nanomaterials-12-03673-s001.zip › nanomaterials-1914053-supplementary.pdf]

SUPPLEMENTARY DATA

Table S1 The crystalline information of each nanoparticle

Sample	Peak position	d-spacing	full width at half maximum (FWHM)	Crystallite size (nm)	Average crystallite size (nm)
CTS-Si/HNT	9.8	0.877	2.82	2.96	3.15
	17.78	0.438	1.617	5.20	
	34.24	0.454	6.75	1.29	
HNT	11.72	0.749	1.183	11.14	18.37
	20.23	0.469	0.899	17.98	
	34.86	0.335	2.01	25.98	
CTS-Si	9.54	0.961	3.52	8.67	13.74
	19.80	0.448	1.37	18.82	

Table S2 TGA data of CTS-Si/HNT nanocomposite in air

Nanocomposite	5% degradation temperature (°C)	10% degradation temperature (°C)	Structural dehydroxylation temperature (°C)	Maximum weight loss onset temperature (°C)
HNT	342	400	420	730
Chitosan	167	313	600	700
CTS-Si/HNT	384	487	570	740
